# From subordination to complementarity?*

**DOI:** 10.1590/1518-8345.0000.3355

**Published:** 2020-08-12

**Authors:** Gilles Dussault

**Affiliations:** 1 Universidade NOVA de Lisboa, Instituto de Higiene e Medicina Tropical, Global Health and Tropical Medicine, Lisboa, Portugal.


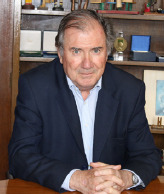
 In 2020, two series of events highlighted the centrality of the role of nurses in the delivery of health services; one was planned, the other not. First, there is the designation by the World Health Assembly of 2020 as the International Year of the Nurse and the Midwife and the publication by World Health Organization (WHO), with the support of the International Council of Nurses and of the *Nursing Now* campaign, of the *State of the World’s Nursing 2020* (SOWN)^(^ 1 ^)^. Then came the unexpected one, the Covid-19 pandemic. Only time will tell the impact of those two events, but we can already identify implications for the education and practice of nurses, the theme of this issue of the Revista Latino-Americana de Enfermagem (RLAE).

The SOWN is the first global portrait of the nursing profession. It documents and discusses the availability and distribution of nurses worldwide, their roles, their working conditions and the quality of their education. While the overall image of the nursing workforce is positive, the report identifies shortcomings that need the attention of the profession and of policy makers. Two examples are the diversity of what is called a “nurses”, and the variations in the density of nurses by population. The report counted 144 distinct titles of nurses around the world, with differences in competencies requirements, education contents and strategies, and in scopes of practice. In the WHO Region of the Americas, 31 titles were identified, not counting assistants or auxiliaries. Some countries recognize a high degree of autonomy, including prescription rights, to certain categories of nurses, while others still limit the role of nurses to subordination to physicians. A second example is the extreme variations in the density of nurses between regions of the world and between countries of different level of economic development. Among the six WHO regions, the Americas is the Region with the highest density (83.4/10000), higher than Europe (79.3). However, this figure conceals the great differences between countries, ranging from 106 and 111/10000 in Canada and the United State of America (USA) respectively, to less than 4/10000 in Haiti, Honduras and the Dominican Republic with^(^ 2 ^)^.

As to the health crisis, it has revealed the lack of preparedness of countries, even those with abundant resources, like Canada, the USA and a number of European countries, and the lack of solidarity between countries and sometimes within the same country. The warnings and advices of international health authorities, namely WHO, and of national public health ones, have not always been timely accepted by political decision makers, and were even ignored by some who denied the importance of the epidemics. On the positive side, the crisis showed the dedication of health workers in general and particularly of nurses who are at the forefront of the response to the epidemics, often at the expense of their own health and safety. It highlighted the competencies and the indispensable role of nurses, not only in intensive care as rapidly became evident, but at all levels of service delivery. At the same time, the crisis revealed how demanding the work of nurses is, and how difficult their working conditions are.

These two events show that even though nursing has made great progress in the recent past, major challenges remain to ensure that nurses contribute fully to achieving universal health coverage. In a majority of countries of the world, the main challenge is to increase the nursing workforce to a level that makes health services accessible to everyone. This requires at least three changes in the education pipeline: to increase the capacity of admission in education institutions, to attract more young people to nursing, and to harmonize education programs and quality assurance mechanisms. Regarding practice, challenges include to provide nurses with a safe, supportive and motivating work environment and to develop or strengthen the monitoring and promotion of the quality of work .

In order to respond to these challenges, effective health workforce policies focusing on education are needed. In nursing, this means investing in the capacity of education institutions and in the recruitment of more educators; also in measures to attract more suitable candidates, including men, to nursing studies, and to prevent attrition during studies so that most admitted students actually graduate. However, more does not mean more of the same: nurses must be equipped with the right competencies, in alignment with the needs of health services and with those of the population^(^ 3 ^-^ 4 ^)^ including in areas that tend to be less valued, such as mental health, geriatrics or primary care. Then all nurses should have the opportunity to adapt and improve their competencies all along their career. The harmonization of programs and of their quality can be realized through the accreditation, by independent bodies, of schools, whether public or private, and of pre-service, clinical training, and long-life programs^(^ 5 ^)^. This would facilitate the harmonization of titles, including of specialties, though, in the context of a globalized health labour market, countries must offer employment and working conditions that encourage retention and limit emigration of the nurses they train.

Scaling-up the status of nurses will enable them to contribute more fully to the availability and quality of health services, in roles that are complementary to that of other health professionals. This is challenging in an environment in which numerous actors with competing interests and objectives interact. For-profit private schools may resist regulation, or medical organizations may oppose the expansion of the role of nurses. Advocacy and recommendations by international agencies like WHO and the Pan American Health Organization (PAHO), and by international and regional professional associations, can help mobilize the political support needed for change to happen. Research is also important to inform policy decisions, and, in that respect, nursing schools and faculties play a critical role in producing the evidence that can convince political decision makers to invest in nursing education and in the development of the profession.

This thematic issue of the RLAE is therefore more than opportune, as it stimulates the dissemination of research results and of innovative actions within the profession. It will show that nursing can move from roles of subordination to ones of complementarity, and make a difference to the benefit of everyone. The next step is to translate this message in a language that speaks to policy makers and to the population, who will be the ultimate beneficiary of a stronger and better performing nursing workforce.
